# An analysis of single amino acid repeats as use case for application specific background models

**DOI:** 10.1186/1471-2105-12-173

**Published:** 2011-05-19

**Authors:** Paweł P Łabaj, Peter Sykacek, David P Kreil

**Affiliations:** 1 Chair of Bioinformatics, Boku University Vienna, Muthgasse 18, 1190 Vienna, Austria

## Abstract

**Background:**

Sequence analysis aims to identify biologically relevant signals against a backdrop of functionally meaningless variation. Increasingly, it is recognized that the quality of the background model directly affects the performance of analyses. State-of-the-art approaches rely on classical sequence models that are adapted to the studied dataset. Although performing well in the analysis of globular protein domains, these models break down in regions of stronger compositional bias or low complexity. While these regions are typically filtered, there is increasing anecdotal evidence of functional roles. This motivates an exploration of more complex sequence models and application-specific approaches for the investigation of biased regions.

**Results:**

Traditional Markov-chains and application-specific regression models are compared using the example of predicting runs of single amino acids, a particularly simple class of biased regions. Cross-fold validation experiments reveal that the alternative regression models capture the multi-variate trends well, despite their low dimensionality and in contrast even to higher-order Markov-predictors. We show how the significance of unusual observations can be computed for such empirical models. The power of a dedicated model in the detection of biologically interesting signals is then demonstrated in an analysis identifying the unexpected enrichment of contiguous leucine-repeats in signal-peptides. Considering different reference sets, we show how the question examined actually defines what constitutes the 'background'. Results can thus be highly sensitive to the choice of appropriate model training sets. Conversely, the choice of reference data determines the questions that can be investigated in an analysis.

**Conclusions:**

Using a specific case of studying biased regions as an example, we have demonstrated that the construction of application-specific background models is both necessary and feasible in a challenging sequence analysis situation.

## Background

In the post-genomic era, with the abundance of sequencing data, the functional interpretation of these sequences constitutes a key challenge. In particular, the identification of biologically relevant differences or shared patterns against a backdrop of functionally meaningless variation is of interest. In computational sequence analysis, this corresponds to detecting unusual patterns relative to a 'background' model [1-3]. The quality of this model directly affects analysis performance. Tools for homology detection by sequence similarity, like FASTA [4] or BLAST [5-7], or the identification of functional sites by pattern conservation in multiple sequence alignments [8,9] traditionally assume positional independence of residues. More recent approaches, including Hidden-Markov-Models (HMMs) [10,11], allow a local dependency structure. In either case, proteins are considered as 'slightly edited random sequences' [12]. Indeed, the complexity of protein sequences reaches 99% of the maximum possible complexity (complete randomness) [13]. Amino acids can apparently often be exchanged for alternatives with similar physicochemical properties [14,15], with the exception of key residues, such as those contributing to an active centre [16].

Interestingly, the same models are typically used for different types of analysis and for all the sequences studied. Increasingly, however, the power and advantages of adjusting background models so that they explicitly take the nature of the studied data sets and/or the question at hand into account are being recognized. One of the first such applications of background-HMMs tailored for the detection of selected functional domains was introduced by PFAM [11]. Recent statistics assessing sequence similarity now can adjust the expected frequencies for each protein [17]. On the other hand, problem-specific background models have been developed, where *e. g*., advanced secondary-structure background models are used for scoring multiple alignments [18].

While standard background models in general perform well in the analysis of globular protein domains, they are known to break down in regions of stronger compositional bias or low complexity, giving nonspecific false positives. In general, these regions are not conserved, and are therefore assumed to be tolerated as neutral and consequently filtered and excluded [19,20]. In contrast, accumulating evidence suggests functional roles of biased regions [21-23], as is for example reflected in the involvement of single amino acid repeats (SAARs) in a number of diseases [24-26]. Furthermore, SAARs can also be an important factor in transcriptional regulation [27,28] and protein interaction networks [29], affect morphological changes [30], and may facilitate adaptive processes [31,32]. As a result, the study of these particularly simple amino acid repeats is attracting increasing interest, despite the difficulty of detecting biased regions of potential biological relevance [22,23].

With the unexpected abundance of single amino acid repeats, detection of those with potential biological function is particularly challenging. As the observed frequency of SAARs cannot be captured by standard models [33-35], an exploration of more complex approaches becomes necessary. We here introduce and validate an empirical background model constructed specifically for this application, which adapts to the characteristics of the studied data set. Our approach is then demonstrated on a practical use case. We thus demonstrate how alternative, appropriate background models can be constructed successfully also in challenging cases. These methods are directly applicable to more general related questions regarding biased or low-complexity regions, while similar empirical constructions will be helpful in other situations in which the established standard models break down.

## Results and Discussion

In a comparison of the three kingdoms [36], bacteria and archaea typically had much fewer single amino acid repeats (SAARs) than eukaryotes. Consequently our analysis focused on eukaryotes.

### Model choice

As the standard sequence model of positional independence is, in fact, a Markov model of order zero, higher order Markov models were obvious candidates for an attempt to capture more complex structures in protein sequences. Prediction performance was assessed for SAARs of length five in a comprehensive set of non-fragmented eukaryotic proteins. Statistically, under the standard model, repeats shorter than five are already expected by random chance for typical protein lengths [37]. Incidentally, five residues is also the shortest repeat-length that has been implicated in diseases [24]. Markov models of orders zero to three were considered, including the most complex Markov model that is meaningful for this pattern length (Suppl. Table S1 in Additional File 1).

**Figure 1:**
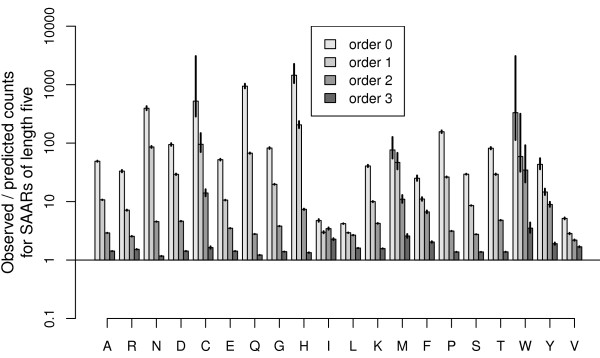
**Comparison of Markov models**. For each amino acid (*x*-axis), the bars present the ratios of observed *vs *predicted counts of repeats with length five. Model standard deviations are indicated by whiskers. Predictions for the standard sequence model of positional independence (a Markov model of order zero) are compared to predictions from higher order models (bar shading), including the highest order model possible for this repeat length. Higher order models yield ratios closer to one, which represents the perfect agreement of observation and prediction.

Figure 1 plots the ratio of observed and predicted SAAR counts (*y*-axis) for all amino acids (*x*-axis), comparing the Markov models of different order (bar shading). Whiskers indicate the model standard deviations. As expected, SAAR abundance cannot be explained by the standard model assuming positional independence of amino acids. It is striking, however, that higher order models also do not capture the observed behaviour. Even for relatively short SAARs, significantly more repeats were observed than predicted. While deviations decreased with increasing model order, even at the highest meaningful model order for SAARs of length five, predictions were systematically too low, with over 50% more repeats observed than predicted (*p *< 10^-35^). Moreover, prediction performance deteriorated further with increasing SAAR length (Suppl. Fig. S1 in Additional File 1). It therefore seems that this popular model class, which predicts the occurrence of longer features from short local dependency structures, cannot be used to describe SAAR frequencies.

Non-local models of repeats are apparently required. The parameters of such models have to be learned from multiple sequences, which will differ in amino acid composition and length. We know that both of these affect the expected number of repeats. Protein sequences, however, do not uniformly occupy the corresponding high dimensional parameter space. Moreover, while repeats in general are unexpectedly frequent, they still constitute relatively rare events, with many proteins not containing repeats at all. For investigations of general functional associations of repeats, one wants a model that has balanced positive and negative errors for realistic sets of proteins. To this end, models can directly be tuned for the correct prediction of repeats observed in the tested protein sets. Optimizing average prediction performance for groups of proteins may moreover allow simpler models of satisfactory quality.

**Figure 2:**
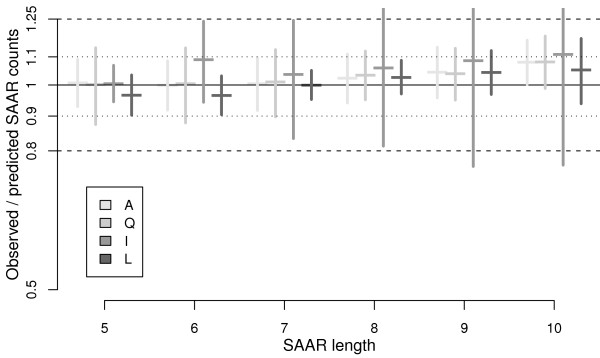
**Model fit quality assessed for the zero-inflated RVM**. We plot the ratio of observed and predicted counts (*y*-axis) for repeats of lengths five to ten (*x*-axis). The horizontal whiskers represent the mean values for a selection of amino acids (A, Q, I, and L, as indicated by shades of grey). The vertical whiskers indicate uncertainty, showing the model standard deviation. More than 90% of all observations fall within 10% of the prediction (dotted horizontal lines). The dashed horizontal lines represent deviations by 25%. Comprehensive graphs for all amino acids are provided by Suppl. Fig. S2 in Additional File 1.

**Figure 3:**
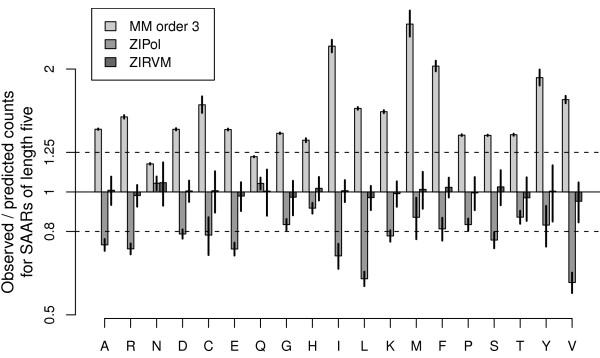
**Comparison of model fit**. For each amino acid (*x*-axis), we compare the three best models (bar shading): a zero-inflated RVM (ZIRVM), a zero-inflated polynomial model (ZIPol), and a third order Markov model (MM). The bars show the ratios of observed and predicted counts for repeats of length five (*y*-axis). The model standard deviations are indicated by whiskers. The dashed horizontal lines represent deviations by 25%, facilitating comparisons with Fig. 2. The base line at the value of one represents perfect agreement of prediction and observation. It lies within less than one standard deviation of the ZIRVM model prediction for all amino acids.

In order to allow comparative analyses of SAAR abundance, we introduce and validate a dedicated zero-inflated relevance vector machine model (ZIRVM). First, the probability that a given protein has at least one repeat is assessed. Then, the conditionally expected number of repeats is modelled by an RVM. Figure 2 plots the ratio of observed and predicted counts (*y*-axis) for repeats of lengths five to ten (*x*-axis). The horizontal whiskers show the mean values for a selection of amino acids (A, Q, I, and L, as indicated by the legend). The vertical whiskers represent the prediction uncertainty. Comprehensive graphs for all amino acids are provided in Suppl. Fig. S2. The typical deviations from the model prediction were less than 2% across all amino acids and SAAR lengths, with more than 90% of all observations being within 10% of the prediction (dotted horizontal lines). In contrast to the Markov models, which vastly under-predicted the occurrence of longer repeats (Suppl. Fig. S1), the introduced ZIRVM captured the observed dependencies of the repeat frequencies on the repeat length. Similarly, the observed dependencies on the other model parameters - the amino acid composition and the protein length - were traced well by the prediction (Suppl. Fig. S3 and Fig. S4). The complementary Suppl. Figs can be found in Additional File 1. Despite its better performance, strikingly, the combined model is three orders of magnitude less complex than the best performing Markov model. We were therefore also interested in examining an even simpler application specific approach, where a standard polynomial fit replaces the more powerful RVM, giving a zero-inflated polynomial model (ZIPol). Figure 3 compares the three models (bar shading) for each amino acid (*x*-axis), showing the ratios of observed *versus *predicted counts of repeats of length five (*y*-axis). Model standard deviations are indicated by whiskers. Both application-specific models outperformed the Markov model, with the ZIRVM model being the overall winner by a clear margin. In particular, the value of one, representing perfect agreement of prediction and observation, lies within less than one standard deviation of the ZIRVM model prediction for all amino acids.

Similarly when considering, for each amino acid, the mean prediction error (RMSE) for all repeat lengths, both application-specific models did much better than the Markov model (Suppl. Fig. S5A). It is noteworthy that even the simple application-specific ZIPol model easily outperformed the more complex Markov model. Examining predictions for individual amino acids and repeat lengths, statistically significant deviations from the model were observed for 81% and 60% of the Markov and ZIPol predictions, respectively. Both models performed worse in the apparently harder prediction of frequent longer repeats. In contrast, no significant deviations were observed for the ZIRVM model at all (Suppl. Fig. S5B). Consequently, protein sets with typical repeat frequencies can be used as reference sets with the application-specific models. See Additional File 1 for Suppl. Figs.

### Use case: SAARs in signal peptides

In order to demonstrate the benefits of a dedicated background model, we apply the introduced application specific model in an analysis of the over-representation of single amino acid repeats in signal peptides. The amino-end of the growing polypeptide chain of secreted and many membrane proteins contains a signal peptide, with a central part rich in hydrophobic amino acids. It has been observed that the location of many SAARs shows a positional bias towards the termini of polypeptides [34,38-40]. Although a possible association of leucine repeats with signal peptides has been suggested earlier [34], there has been no systematic study of repeat enrichment in signal peptides. In particular, a quantitative test is not possible without an appropriate background model to account for the effects of varying amino acid composition and sequence lengths on the observed repeat counts.

We now consider how application specific background models enable quantitative studies, and how the models can adapt to address specific questions, also adjusting to interim results as an analysis progresses. In the first investigation phase of this use case, a ZIRVM background model was trained on a comprehensive set of proteins without signal peptides and signal anchors. This allows an examination of

**Hypothesis (1) **Mature sequences of secreted and type I membrane proteins do not differ regarding the distribution of repeats from proteins with no signal sequence.

A model of the repeat distribution in proteins without a signal peptide would thus also capture the observed frequencies of repeats in the mature parts of proteins that had that transient peptide cleaved off. If this hypothesis holds - *i. e*., a common model can be learned from the comprehensive set of proteins with no signal sequences - then we can formulate the central question of the use case as follows:

**Hypothesis (2) **Signal peptides show an unusual enrichment of certain repeats (relative to a common background distribution).

The 'Null Hypothesis' expectation for the first test was that the ratio of observed and predicted repeat counts in the mature sequences would not be significantly different from the distribution of this ratio for proteins without signal peptides. Although this was indeed the case for longer repeats of the three strongly hydrophobic amino acids F, I, and V, surprisingly, highly significant differences were observed otherwise, affecting more than 80% of all amino acid/repeat length combinations (Suppl. Tab. S2 in Additional File 1). As a result we can strongly reject hypothesis (1).

The observation that the mature parts of secreted and type I membrane proteins have a distinct repeat distribution is actually interesting in its own right. Furthermore, this result changes the kind of related questions that we can ask and how we can test them, highlighting the need to first explicitly validate the (sometimes implicit) underlying assumptions of hypotheses. In particular, we now know that there is no common background distribution, making the original, simple formulation of the second hypothesis void. Background models fitted on proteins without signal peptides are in general not appropriate for examining a potential enrichment of repeats in signal peptides relative to the mature parts of the protein.

Such an analysis becomes possible after adapting the background model. By training on mature sequences of secreted and type I membrane proteins we can test the use case question as

**Hypothesis (2b) **Signal peptides show an unusual enrichment of certain repeats sequences relative to the mature parts.

**Figure 4:**
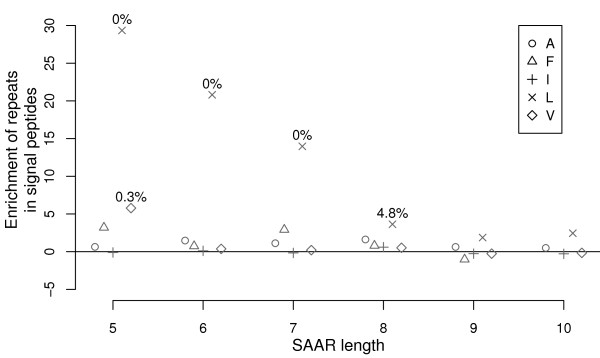
**Enrichment of single amino acid repeats in signal peptides**. We compare the SAAR frequency in the whole protein (including the signal peptide) to the SAAR frequency in the mature parts. For a standardized display, we consider ratios of observed SAAR counts versus counts predicted by the background model. We plot the difference between these ratios for whole and mature proteins in terms of model standard deviations (*y*-axis), and test for positive values. For significant enrichment (*p *< 5%) the *p*-values are printed next to the symbol. Median data are shown for a selection of hydrophobic amino acids (A, F, I, L, and V, as indicated by symbols) and repeat lengths from five to ten (*x*-axis).

Interestingly, a strong, significant over-representation was only found for leucine repeats, but not for any of the other hydrophobic amino acids abundant in signal peptides: Figure 4 plots a measure of the enrichment of repeats in signal peptides. For this we compare the SAAR frequency in the whole protein (including the signal peptide) to the frequency in the mature parts, for repeat lengths from five to ten (*x*-axis). In order to see results for different amino acids on a comparable scale, we consider ratios of observed SAAR counts *versus *counts predicted by the background model. If there is no enrichment, these ratios will be one, both for the whole protein and the mature parts. Any deviation for the whole protein indicates indicates over- or under-representation of SAARs in the signal peptide. We therefore plot the difference between the ratios for whole and mature protein in terms of model standard deviations (*y*-axis), and test for enrichment (*p *< 5%, as displayed in the graph).

While the figure examines the hydrophobic residues most frequent in signal peptides (A, F, I, L, and V, as indicated by the legend), we here actually report results for all amino acids (Suppl. Tab. S3 of the Additional File 1). It is noteworthy that in a complete survey of all eukaryotic proteins for SAARs of all amino acids there was no trend for enrichment of repeats in signal peptides other than the reported general significant overrepresentation of leucine repeats. This was unexpected, considering that the also hydrophobic alanine is similarly abundant in the core region of signal peptides and that the related hydrophobic amino acids isoleucine and valine are also frequent there. While it is understood that this hydrophobic region is required for interaction with the signal recognition particle, recent work has uncovered a surprising complexity of signal sequences [41] and suggested additional functions such as in the modulation of protein biogenesis [42]. The unusual enrichment of leucine repeats in signal peptides of eukaryotes may thus serve another purpose, although their exact role yet remains to be investigated by experiments.

## Conclusions

Recent developments in sequence analysis have made it increasingly apparent that empirical adjustments or novel application-specific approaches are required to define a suitable baseline for each study. In particular, the identification of biologically relevant differences or shared patterns - against a backdrop of functionally meaningless variation - corresponds to identifying unusual observations relative to an appropriate 'background' model. The quality of the background model directly affects the performance of an analysis. For example, adjustments of statistics for protein amino acid composition have considerably improved existing sequence analysis tools for homology detection [17]. Still, regions of stronger compositional bias are traditionally filtered because they lead to a localised breakdown of the classic background model, making them more difficult to study quantitatively [19,20].

In this manuscript, we have explored the suitability of more complex sequence models and application-specific approaches for the investigation of biased regions, using specific sequence repeats as example. Interestingly, even the most complex local sequence models could not predict the high frequency of the observed repeat regions. In contrast, application-specific zero-inflated models consistently performed better, despite their much lower dimensionality. In particular, we could show that a zero-inflated RVM model (ZIRVM) captured the multi-variate dependencies well. It was also flexible enough for application in different scenarios, adapting to the reference data and question at hand.

These observations are, moreover, of wider relevance: Biased regions are abundant in most organisms. Although not conserved in general, certain biased regions are increasingly implicated in functional roles [21-23]. For traditional sequence similarity based methods of homology detection in the study of protein structure and function, however, biased regions have to be filtered. In contrast, an observed significant enrichment of biased regions in certain protein classes or selected sequence parts can aid functional comparisons at the feature level [43]. We have here chosen to study single amino acid repeats (SAARs), which form a particularly simple class of biased regions. Nevertheless, their high frequency and their potential functions are not fully understood [34]. SAARs have, however, anecdotally been identified as causing a number of diseases [24-26], constituting an important factor in transcriptional regulation [27,28] and protein interaction networks [29], affecting morphological changes [30], and contributing to the facilitation of adaptive processes [31,32].

In our use case for the application specific model, we identified general differences in the repeat distribution between proteins without a signal peptide and the mature parts of proteins remaining after cleavage of these transient regions. Considering this evidence of the heterogeneous nature of protein space, we next focused on proteins with signal peptides, adapting the background model accordingly. Relative to the mature sequences, further investigation identified leucine repeats as highly enriched in eukaryotic signal peptides, in contrast to repeats of any other amino acid. This is remarkable because it sets leucine apart from the remaining hydrophobic residues frequently found in signal peptides. As we have shown in a study of smaller scope elsewhere, these repeats are actually better conserved than their surrounding host sequence, suggesting a yet unknown function [36]. We have shown here that this is unique to leucine and that no other amino acids exhibit a similar trend for significant enrichment in signal peptides.

To summarize, in challenging sequence analysis situations the construction and validation of appropriate background models can become necessary. Using a specific case of studying biased regions as an example, we have shown the breakdown of both the standard sequence model of positional independence and higher-order local predictors. We have then illustrated the usefulness of dedicated, application-specific background models in the detection of biologically interesting signals. Besides highlighting the importance of selecting a suitable background model, our use case also shows how the question examined actually defines what constitutes the 'background', and thus determines an appropriate reference training set.

## Methods

### Data and feature extraction

Primary sequence data and analysis results were managed in a customized InterMine data warehouse [44,45]. Protein sequences were obtained from UniProt [46] release 15.13. To minimize artefacts, proteins annotated in UniProt as fragments were filtered because they could particularly affect signal peptide prediction, where knowing the start of the protein sequence is important. As SAARs are rare in *Bacteria *and *Archaea *[36] we concentrated on eukaryotic proteins, yielding a total of 1.9 million sequences. To facilitate the model fit, we construct a function from the counts of non-overlapping single amino acid tracts. The cumulative distribution of tract counts, in particular, provides an exact measure of repeat abundance while being easier to model than the tract counts themselves as it has a smaller number of jumps. Consequently, this is the function that is being modelled in this paper, and when we report 'repeat counts', we are referring to these cumulative counts. For example, in the sequence ACDFLLLLLGWSLLV there is one non-overlapping leucine tract of length five and one non-overlapping leucine tract of length two. Constructing the cumulative count distribution, for this example sequence, we observe one SAAR of length five (or longer), one SAAR of length four or longer, one of length three or longer and two SAARs of length two or longer. It is these cumulative counts that we report, often dropping the implied 'or longer' in the manuscript text.

SAARs were identified and counted by custom Perl scripts. Feature statistics were computed in the 'R' statistical environment [47]. Considering that frequencies were clearly residue specific, we model repeat frequencies independently for each amino acid.

### Traditional sequence models

The challenge of identifying biologically relevant patterns is central to sequence analysis in bioinformatics and, consequently, a variety of complementary tools exist to detect unusual sequence regions. An established generic and powerful class of models captures local dependencies by way of Markov models, as supported, for example, by RSAT [3,48], R'MES [49], QuickScore [50], and SPatt [51]. Different implementations have their own respective strengths and features, including providing a service online with user support, optimized run-time performance, and different statistical approximation options for the assessment of significance. SPatt, in particular, offers statistics that are suitable for a direct comparison to traditional, non-overlapping pattern counts, and was therefore employed for our analyses. SPatt 2.0 was run with default parameters for Gaussian approximations, testing Markov models of orders zero to three. This includes both the highest meaningful model order for patterns of length five as well as the standard model of positional independence, which is a Markov model of order zero (also see Figure 1). 

Parameters were obtained from the respective *k *- tuple frequencies (*k *= 1 ... 4) of the comprehensive set of non-fragmented eukaryotic proteins from UniProt (about 10^9 ^residues). Model complexity thus ranged from 20 parameters for the positional independence model to 20^4 ^= 160,000 parameters for the third order Markov model.

### Application specific models

For capturing both the exponential scale behaviour of the frequency of the repeats as a function of their lengths, but also the occurrence of proteins with none at all, a zero-inflated model was found to be efficient: First, logistic regression was used to give the probability that a protein of given amino acid composition and length had at least one repeat of a particular residue type and repeat length. Then, a relevance vector machine [52] predicted the conditionally expected number of repeats as a function of repeat length, residue composition, and protein length, each on a logarithmic scale. Here, the logistic regression step in particular captures the considerable number of proteins with zero repeats (hence 'zero-inflated'). All regression and model testing was performed in the 'R' statistical environment. The standard function for fitting a generalized linear model (glm) was used for logistic regression. The relevance vector machine was trained using the rvm function of the established kernlab library [53]. The zero-inflated relevance vector machine (ZIRVM) model had a complexity of 900-2,100 parameters depending on the random starting point of the algorithm and the complexity of the training data set. For the evaluation of a simpler application specific approach, in the second stage, a polynomial model was fitted instead of an RVM,

where *c *is the modelled count of repeats of non-overlapping *n *amino acids of residue type *AA*, conditional on the predicted existence of any such repeats in a protein of length *l *with a given amino acid composition *f_AA_*. Coefficients *x_i _*were obtained by standard least-squares fit using the nls function. This zero-inflated polynomial (ZIPol) model had a total of 304 parameters.

#### Model validation

For each protein, amino acid residue, and repeat length, the number of SAARs was recorded, giving a total of about 350 million observations. Besides the repeat length, the application specific models also considered protein length and amino acid frequencies as covariates to the SAAR counts.

For each residue type, a separate predictor was trained in a two step process: First, about three million data points were subsampled for logistic regression. Classification thresholds were empirically chosen to strike an equal balance between positive and negative prediction errors for each repeat length.

For the second modelling step, weighted subsampling was used to correct for the highly skewed nature of the data: Much fewer repeats were observed for higher repeat lengths, or at lower amino acid frequencies. For example, there were 13,675, 4,357, 1,642, 642, 294, and 145 R-repeats of lengths five to ten. Training sets were therefore compiled that balanced the number of observations in bins of defined protein lengths, amino acid composition, and repeat length. The total number of data points subsampled was limited to 2,000 for practical reasons in the RVM training.

Model quality was verified in 100-fold cross-validation, with two thirds of the comprehensive set of non-fragmented eukaryotic proteins used for training, and the remaining third as independent test set. For each repetition, we computed the ratio of observed and predicted repeat counts as a test score. We assess model fit by comparing the distribution of test scores to the perfect score of 1, calculating an empirical *p*-value. In plots, the standard deviation of this distribution is shown to indicate the prediction model uncertainty. Capturing average performance for each amino acid, we calculate the root mean square error (RMSE) over all repeat lengths. Calculations were performed on a log-scale for symmetrical weighting of over- and underprediction.

For all residue types except tryptophan both ZIRVM and ZIPol models could be fit and verified in 100-fold cross-validation. Longer tryptophan repeats are extremely rare, which makes an independent validation of the model fit difficult.

This validation demonstrates the ability of a model to capture the relevant sequence properties in a comprehensive set of proteins, thus identifying a relevant sequence model for the data examined. With an appropriately chosen reference set, it can serve as a background model.

### Use case

To demonstrate the benefits of introducing application specific background models we explored the abundance of repeats in signal peptides.

For a conservative prediction of signal peptides, we combined the neural network and Hidden-Markov-Model predictors of SignalP 3.0 [54,55] applied using their default settings. Predictions were accepted if both methods agreed on the location of the cleavage site, at least one of the prediction scores met its default threshold, and the worse score reached at least half its threshold. Combining both predictors exploits the high sensitivity of the neural network in the detection of signal peptides, while still allowing a discrimination of signal anchors by the Hidden-Markov-Model component [56].

In the first phase of this analysis, a ZIRVM model was trained on the non-fragmented eukaryotic proteins having neither a signal peptide nor a signal anchor. We then studied the ratios of observed *vs *predicted SAAR counts for the mature sequences of secreted and type I membrane proteins. Deviations from the ZIRVM background model were assessed by empirical *p*-values computed from the distribution of test scores compiled as before. The reported two-sided test significance of differences for each amino acid is after Holm correction for testing multiple repeat lengths.

For a valid test of repeat enrichment in the signal peptide relative to the mature sequence, in the second phase of the analysis, the mature sequences of secreted and type I membrane proteins were used to train the background model. Testing for enrichment, reported significances are for one-sided tests.

Note that a separate assessment of model fit by cross-validation for the alternative training sets gave similar results to our model comparison on the comprehensive set of proteins (data not shown).

## Authors' contributions

PPL implemented and performed all analyses, including the model construction, selection, and validation, and the use-case study. PS supervised the model construction. DPK conceived the study, supervised model selection, validation, and application to the use case. PPL and DPK wrote the manuscript. All authors have read and approve of the final text.

## Supplementary Material

Additional file 1 **Online Supplement**. We provide an online Supplement with further supporting tables and figures, and also linking to the data and analysis code used for this study. Click here for file
