# Supplementary material for: An analysis of single amino acid repeats as use case for application specific background models
Source: BMC Bioinformatics. 2011 May 19;12:173. doi: 10.1186/1471-2105-12-173 (PMC3124433; doi:10.1186/1471-2105-12-173)
Supplement: Additional file 1 — Online Supplement. We provide an online Supplement with further supporting tables and figures, and also linking to the data and analysis code used for this study. [file 1471-2105-12-173-S1.PDF]

Supplement

Contact by email: repeats10@boku.ac.at
Group webpage: http://www.bioinf.boku.ac.at/

Tables

Residue	Observed count	Counts predicted by Markov models of order zero	one	two	three
A	97660	1992	9114	33546	68553
R	15953	482	2238	6256	10447
N	64082	163	745	14087	54693
D	35522	376	1214	7674	24947
C	757	2	8	54	464
E	47900	920	4521	13678	33688
Q	119444	127	1773	42885	97905
G	71313	867	3609	18734	51217
H	11183	8	55	1515	8353
I	1818	384	609	528	799
L	26415	6301	9021	9909	16490
K	21581	531	2161	5093	13723
M	479	7	11	44	186
F	2279	92	207	342	1121
P	57766	370	2197	18427	41937
S	99002	3366	11552	35897	71947
T	40126	491	1368	8354	29057
W	89	1	2	3	26
Y	944	22	65	106	496
V	3911	760	1378	1784	2324
Training set k-tuples		$k = 1$	$k = 2$	$k = 3$	$k = 4$

Table S1: Observed and predicted repeat counts. Counts of SAARs of length five are shown for eukaryotic proteins as observed (first column) compared to predictions using Markov models of orders zero to three. This includes the standard model (order zero), which is trained on the frequencies of single amino acids (k-tuples with $k = 1$). The most complex Markov model that is meaningful in a prediction of frequency of patterns of five residues is of third order, and needs to be trained on k-tuples of length $k = 4$. All predictions are significantly different from the observed counts, even for the highest order Markov model ($p < 10^{-35}$, Gaussian approximation). The difficulty of a third order model predicting 5-mer SAARs from a 4-mer training set indicates strong non-local effects, with prediction performance decreasing further for longer SAARs (*cf.* Fig. S1). Consequently, although a fourth order model would of course perfectly capture the distribution of 5-mers (on which it is trained), it does not correctly predict the frequency of SAARs of length six (data not shown), *et c.*. In summary, these results demonstrate that the observed SAAR frequencies cannot be explained by traditional local sequence models.

	single amino acid repeat length					
	5	6	7	8	9	10
A						
C			64%	64%		*
D						
E						
F			32%	60%	62%	60%
G						
H						
I	80%		10%		84%	
K			2%			
L	4%			4%		
M				†	†	†
N						
P						
Q						
R					8%	50%
S						
T						
V						
Y						

Table S2: Differences between mature sequences and proteins with no signal peptides. We test the Null hypothesis that the ratio of observed *vs* predicted repeat counts in the mature sequences is not significantly different from the distribution of this ratio in proteins with no signal peptides. Empirical p-values after Holm correction over repeat lengths are shown. Significant values ($p < 5\%$) are marked with a shaded background. Empty fields indicate the extreme values of $p = 0\%$ (shaded) and $p = 100\%$ (white).

* Repeats of a specific length were observed despite a prediction of zero.

† No repeats of a specific length were observed in agreement with the prediction.

	single amino acid repeat length					
	5	6	7	8	9	10
A	67%	42%	52%	42%	67%	67%
C						
D	85%					
E	1%	38%	78%			
F	9%	52%	11%	52%		
G	11%	10%	11%	7%	11%	7%
H	50%	78%	78%	78%	70%	78%
I						
K						
L	0%	0%	0%	4.8%	6%	6%
M	92%	84%	84%	†	†	†
N						
P		48%			71%	
Q	11%	30%	19%	30%	57%	11%
R	8%	54%				
S						
T	85%	50%				
V	0.3%	87%	91%		91%	
Y						

Table S3: Repeat enrichment in signal peptide sequences relative to the mature parts. The Null hypothesis in this test is that the ratio of observed and predicted repeat counts in whole proteins is not significantly different from the background distribution of this ratio (*i. e.*, the distribution in mature sequences). This would imply that the repeat frequency in signal peptides is no different. The alternative hypothesis tested was that this ratio is bigger, implying repeat enrichment. The table displays empirical p-values after Holm correction over repeat lengths. Significant values ($p < 5\%$) are marked with a shaded background. Empty fields indicate $p = 100\%$.

† No repeats of a specific length were observed in agreement with the prediction.

Figures

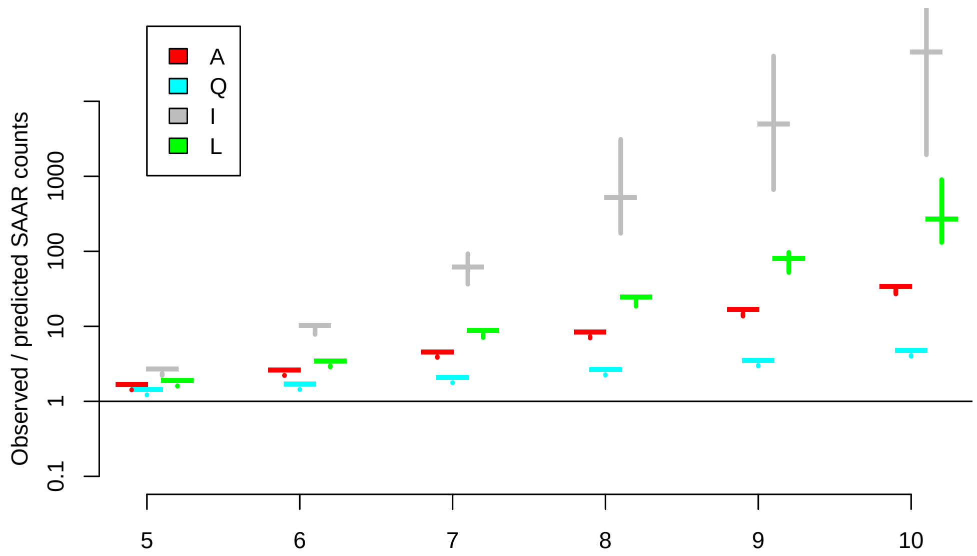

Figure S1: Prediction quality for a Markov Model of order three. We plot the ratio of observed and predicted counts (y-axis) for repeats of lengths five to ten (x-axis). For a selection of amino acids (A, Q, I, and L, as indicated by line colour), the horizontal whiskers represent the mean values, the vertical whiskers the model uncertainty as computed by SPatt. See Suppl. Fig. S6 for plots comparing all amino acids.

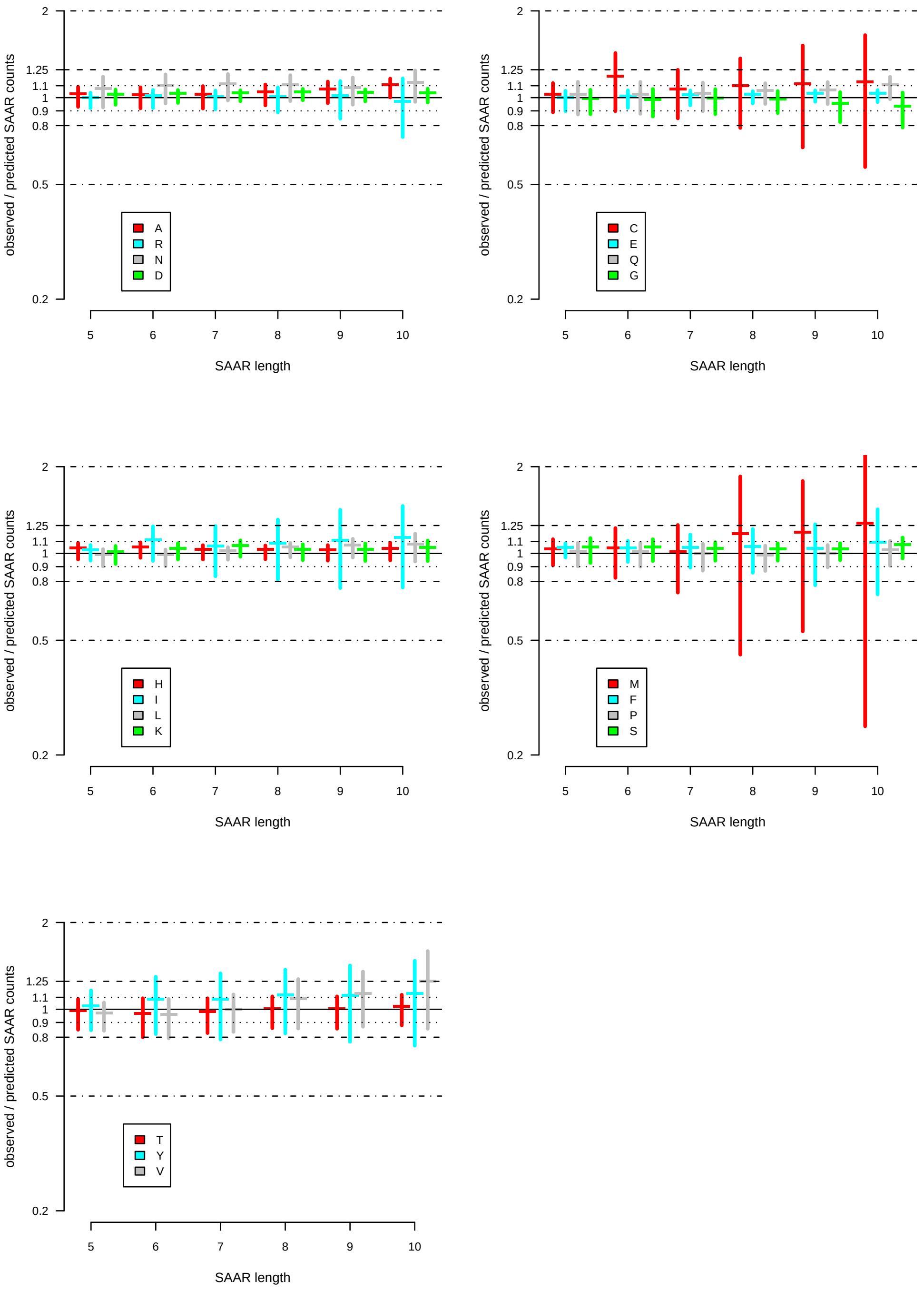

Figure S2: Model fit quality assessed for the zero-inflated RVM, *versus* repeat length as an independent variable. We plot the ratio of observed and predicted counts (y-axis) for repeats of lengths five to ten (x-axis). The horizontal whiskers represent the mean values for all amino acids (four amino acids per plot, as indicated by line colour). The vertical whiskers indicate the model uncertainty. The horizontal markers represent deviations in terms of constant fold-change, facilitating comparisons across panels.

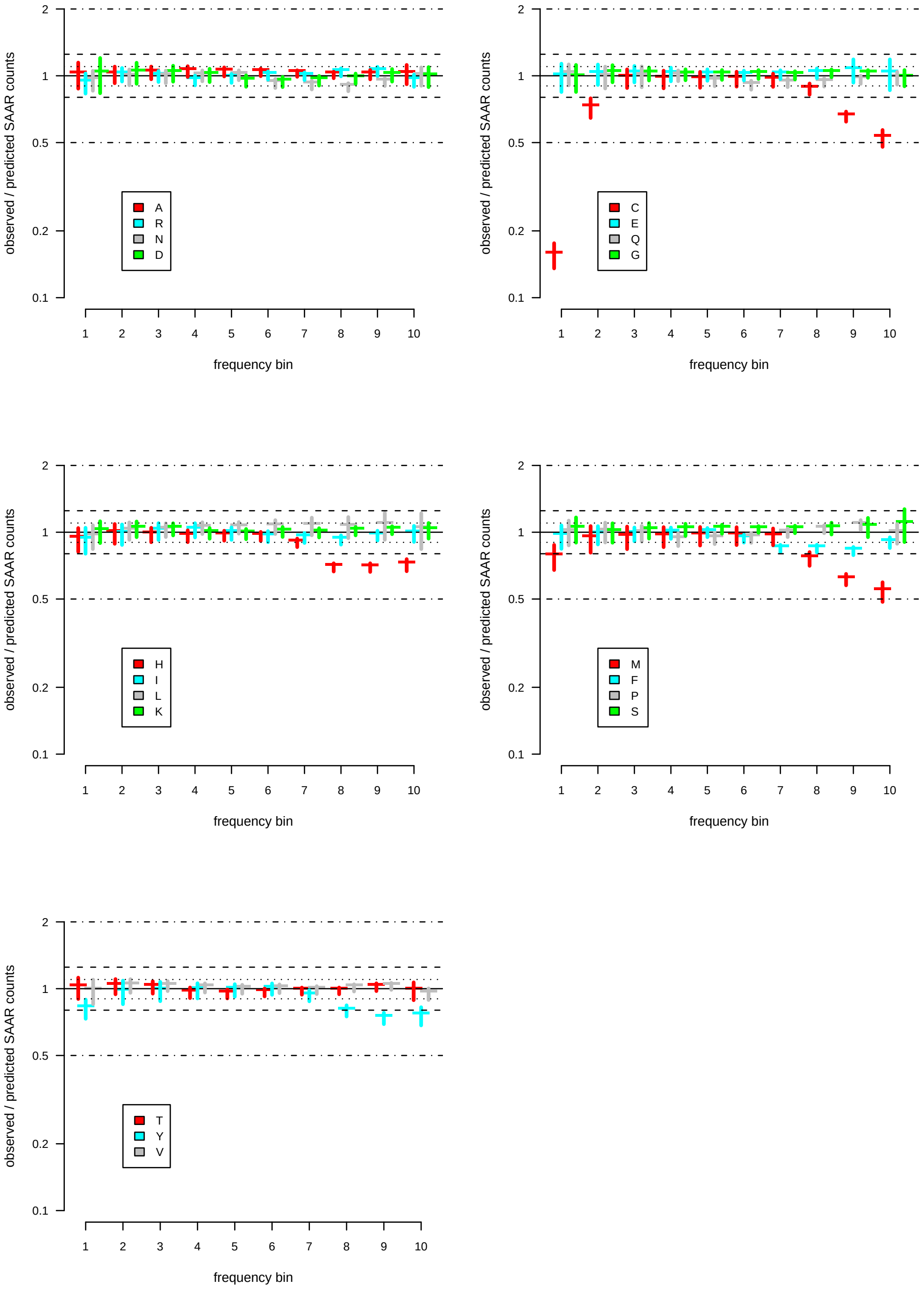

Figure S3: Model fit quality assessed for the zero-inflated RVM *versus* amino acid frequency as an independent variable. We plot the ratio of observed and predicted counts (y-axis) for the whole spectrum of amino acid frequencies (x-axis shows ten-percentile bins). The horizontal whiskers represent the mean values for all amino acids (four amino acids per plot, as indicated by line colour). The vertical whiskers indicate the model uncertainty. The horizontal markers represent deviations in terms of constant fold-change, facilitating comparisons across panels.

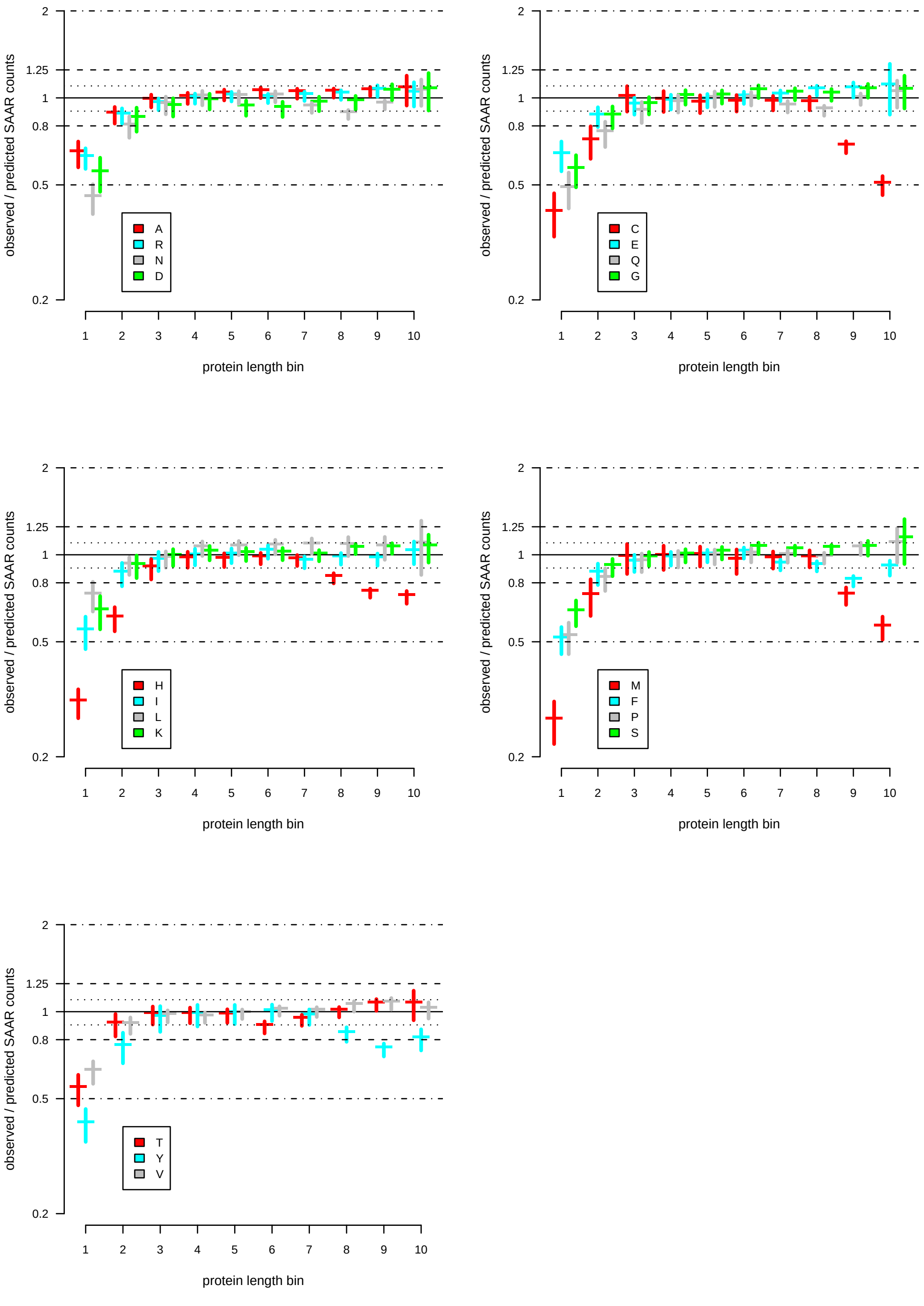

Figure S4: Model fit quality assessed for the zero-inflated RVM *versus* protein length as an independent variable. We plot the ratio of observed and predicted counts (y-axis) for the whole spectrum of protein lengths (x-axis shows ten-percentile bins). The horizontal whiskers represent the mean values for all amino acids (four amino acids per plot, as indicated by line colour). The vertical whiskers indicate the model uncertainty. Although predictions for short proteins are apparently harder and might constitute an area for future improvements, this apparently did not negatively affect the overall model performance Suppl. Fig. S5. The horizontal markers represent deviations in terms of constant fold-change, facilitating comparisons across panels.

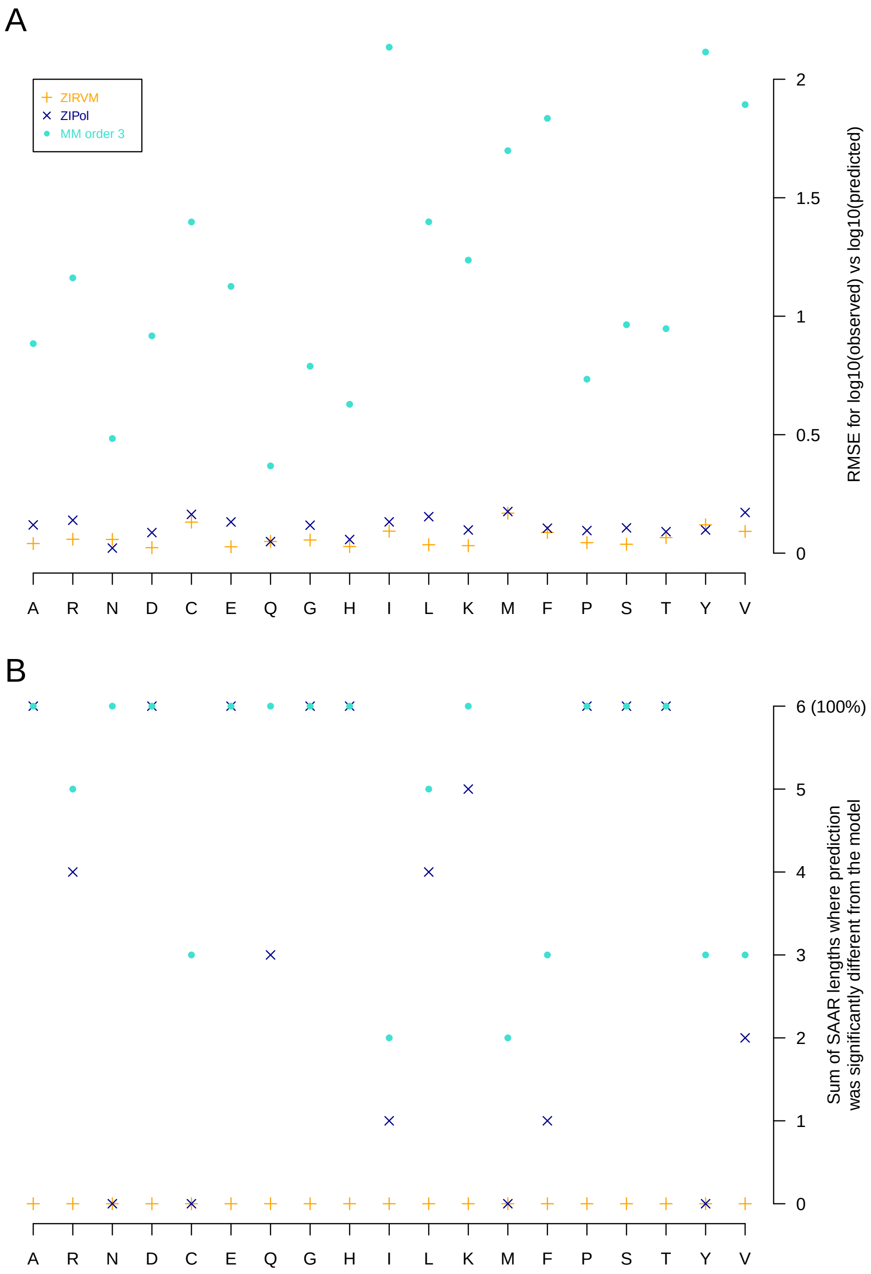

Figure S5: Comparison of the model fit. (A) For each amino acid (x-axis), we plot the mean prediction error (RMSE) for all repeat lengths (y-axis). (B) We plot, for each amino acid (x-axis), the summary over repeat length of statistically significant deviations from the model. A value of six (100%) indicates that, for a given amino acid, for all repeat lengths the prediction was significantly different from the model. Colours and symbols distinguish between the compared models: ZIRVM, ZIPol and Markov model (MM) of order three.

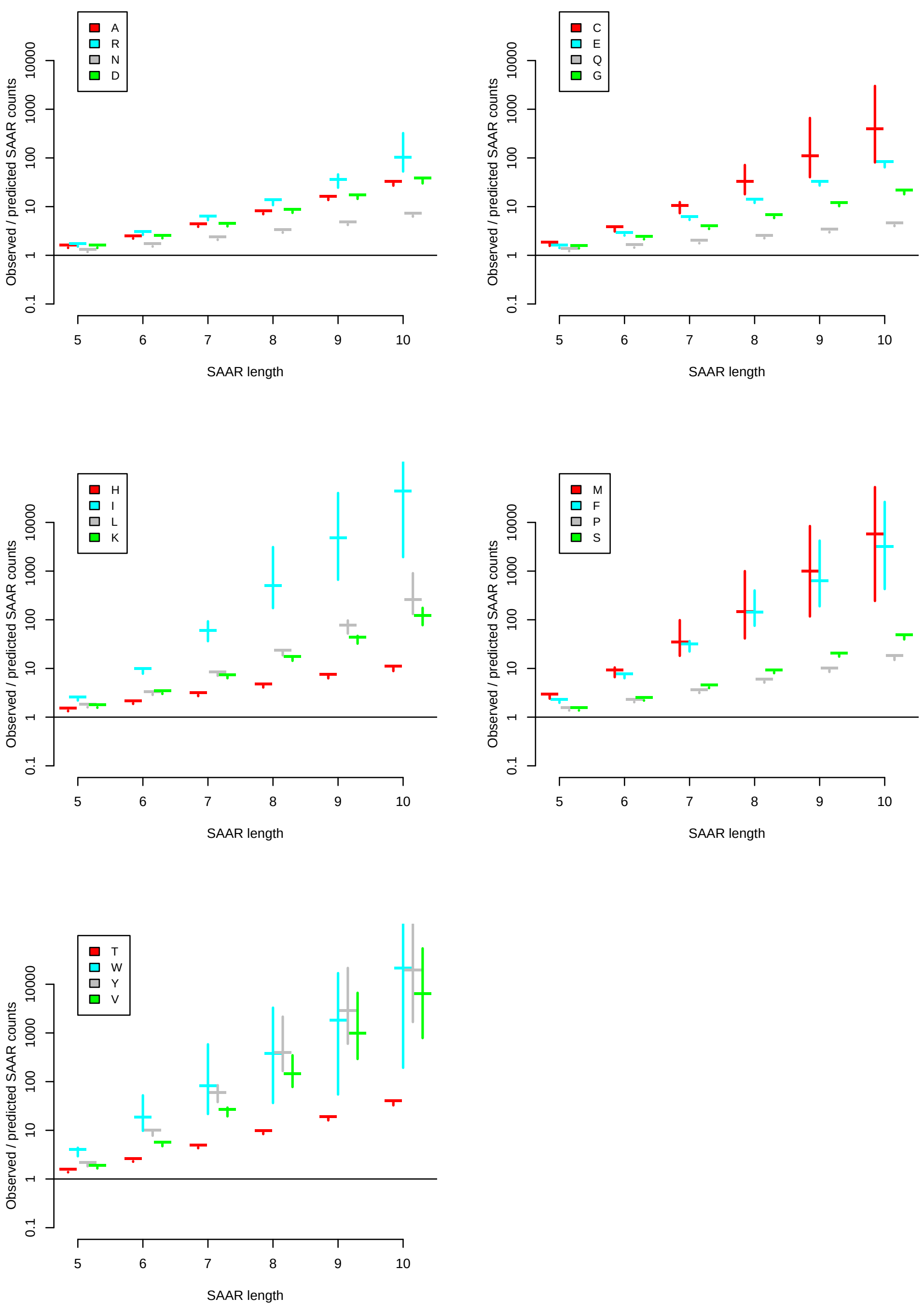

Figure S6: Prediction quality for a Markov Model of order three. We plot the ratio of observed and predicted counts (y-axis) for repeats of lengths five to ten (x-axis). For each amino acid (four amino acids per panel, as indicated by line colour), the horizontal whiskers represent the mean values, the vertical whiskers the model uncertainty as computed by SPatt.

Data and code

We provide:

- The R code for the first phase of the use case analysis at
  http://bioinf.boku.ac.at/pub/aar2010/Hypothesis_1.R.

- The R code for the second phase of the use case analysis at
  http://bioinf.boku.ac.at/pub/aar2010/Hypothesis_2.R.

We also list the corresponding

- input data for the first phase of the use case analysis in `Rdata` format at
  http://bioinf.boku.ac.at/pub/aar2010/Data_1/,

- input data for the second phase of the use case analysis in `Rdata` format at
  http://bioinf.boku.ac.at/pub/aar2010/Data_2/,

for each amino acid. Note that each of the data files is 59–158 MB in size.

References

Information about R and the associated data formats can be found at http://www.r-project.org/.
